# QTL mapping in autotetraploids using SNP dosage information

**DOI:** 10.1007/s00122-014-2347-2

**Published:** 2014-07-01

**Authors:** Christine A. Hackett, John E. Bradshaw, Glenn J. Bryan

**Affiliations:** 1 Biomathematics and Statistics Scotland, Invergowrie, Dundee, UK; 2 The James Hutton Institute, Invergowrie, Dundee, UK

## Abstract

**Key message:**

**Dense linkage maps derived by analysing SNP dosage in autotetraploids provide detailed information about the location of, and genetic model at, quantitative trait loci.**

**Abstract:**

Recent developments in sequencing and genotyping technologies enable researchers to generate high-density single nucleotide polymorphism (SNP) genotype data for mapping studies. For polyploid species, the SNP genotypes are informative about allele dosage, and Hackett et al. (PLoS ONE 8:e63939, 2013) presented theory about how dosage information can be used in linkage map construction and quantitative trait locus (QTL) mapping for an F_1_ population in an autotetraploid species. Here, QTL mapping using dosage information is explored for simulated phenotypic traits of moderate heritability and possibly non-additive effects. Different mapping strategies are compared, looking at additive and more complicated models, and model fitting as a single step or by iteratively re-weighted modelling. We recommend fitting an additive model without iterative re-weighting, and then exploring non-additive models for the genotype means estimated at the most likely position. We apply this strategy to re-analyse traits of high heritability from a potato population of 190 F_1_ individuals: flower colour, maturity, height and resistance to late blight (*Phytophthora infestans* (Mont.) de Bary) and potato cyst nematode (*Globodera pallida*), using a map of 3839 SNPs. The approximate confidence intervals for QTL locations have been improved by the detailed linkage map, and more information about the genetic model at each QTL has been revealed. For several of the reported QTLs, candidate SNPs can be identified, and used to propose candidate trait genes. We conclude that the high marker density is informative about the genetic model at loci of large effects, but that larger populations are needed to detect smaller QTLs.

**Electronic supplementary material:**

The online version of this article (doi:10.1007/s00122-014-2347-2) contains supplementary material, which is available to authorized users.

## Introduction

Much progress has been made in recent years in developing methods for linkage analysis and QTL mapping in autotetraploid species. New genotyping technologies, such as the Illumina Infinium platform and sequencing-based methods, such as RAD sequencing (Baird et al. 2008) or genotyping by sequencing (Elshire et al. 2011), are enabling the construction of high-density single nucleotide polymorphism (SNP) maps. Some of these technologies carry information not simply on presence/absence of an allele at a SNP locus, but about the actual allele dosage, which provides important information in polyploid species.

Hackett et al. (2013) have developed the statistical theory for using SNP dosage information to estimate recombination frequencies between SNPs in an autotetraploid population, and have applied this to a full-sib potato (2*n* = 4*x* = 48) population derived from a cross between processing clone 12601ab1 and the cultivar Stirling. This paper also developed methodology for interval mapping of quantitative trait loci (QTL), using the dosage information. The methodology used a hidden Markov model (HMM) (Rabiner 1989) to estimate genotype probabilities for each offspring along the chromosomes, and then modelled trait values as an additive function of the homologue effects, weighted by the genotype probabilities. This builds on previous work by Hackett et al. (2001) on QTL mapping in autotetraploids, which extended the mixture model approach to mapping QTL of Jansen (1992). The QTL model was applied to analyse allele intensity ratios of SNPs from the Illumina Infinium platform as a check on the position and phase of the SNP markers.

The allele intensity ratios are uncomplicated traits for QTL mapping, in that they are expected to have a very high ratio of genetic to random variance, to map to a single chromosomal location and to follow an additive function of the homologue effects. If a SNP has parental genotypes AABB × AABB, the offspring are expected to have genotypes AAAA, AAAB, AABB, ABBB and BBBB in a 1:8:18:8:1 ratio and the offspring allele intensity ratios are expected to cluster around values of 0.0, 0.25, 0.5, 0.75 and 1.0 (i.e. an additive function of the number of B alleles present). In general, we are interested in mapping traits with lower heritabilities, which may be affected by more than one QTL and where there may be dominance or other non-additive effects.

In this paper, we present a simulation study on the efficacy of QTL interval mapping based on SNP dosages to model more general phenotypic traits of low to moderate heritability. We investigate the threshold LOD score for declaring a QTL present, and compare the additive model with a more complicated model for the 36 possible QTL genotypes. We then apply the model to re-analyse QTL data on this population for flower colour from Bradshaw et al. (2008), for maturity, height and resistance to late blight (*Phytophthora infestans* (Mont.) de Bary) from Bradshaw et al. (2004) and on resistance to potato cyst nematode (PCN, *Globodera pallida)* from Bryan et al. (2004).

## Materials and methods

### Potato mapping population and linkage map

The cross between the potato processing clone 12601ab1 and the cultivar Stirling has been studied extensively, and different linkage maps have been published. An early AFLP map based on 78 F_1_ offspring was published by Meyer et al. (1998) and a more detailed AFLP and SSR map, based on the full population of 227 F_1_ offspring was published by Bradshaw et al. (2008). This map contained 453 mapped markers and identified some, but not all, of the chromosomal groups based on the locations of SSR markers together with shared markers from the ultra high density (UHD) mapping population SH × RH (van Os et al. 2006). Stirling, 12601ab1 and 190 F_1_ offspring have recently been genotyped using the Infinium 8303 potato SNP array (Felcher et al. 2012). A SNP linkage map, using the dosage information, was generated by Hackett et al. (2013). This map assigned locations to 3839 SNPs, and identified all the chromosomal groups. Simplex SNPs identified 46 of the 48 chromosomes for Stirling and 45 chromosomes for 12601ab, giving a very comprehensive linkage map. SNP marker positions in the potato reference genome provided for QTL locations are obtained using the version 4.03 potato genome pseudomolecules (Potato Genome Sequencing Consortium 2011; Sharma et al. 2013).

### Phenotypic traits

In this paper, we re-analyse some phenotypic traits from previous publications on this population by Bradshaw et al. (2004, 2008), Bryan et al. (2004). These papers describe the data collection and preliminary analysis including the estimation of heritability in detail. The following traits were analysed:

Flower colour (Fc), from Bradshaw et al. (2008), scored as a qualitative trait: blue like 12601ab1 or white like Stirling, for the clones that flowered over 3 years of trials (178 out of 190) from 1999 to 2001.

Maturity (Mat), from Bradshaw et al. (2004), scored on a one (early, all plants dead) to nine (late, all plants still green) scale in 1999 and 2000 and analysed as clone means over the 2 years. The heritability was estimated as 0.916.

Canopy height (Ht), from Bradshaw et al. (2004), measured in centimetres (cm) from the top of the drill in 1999, 2000 and 2001 and analysed as clone means over the 3 years. The heritability was estimated as 0.876.

Resistance to late blight (*Phytophthora infestans* (Mont.) de Bary) from Bradshaw et al. (2004), scored as

1. Fourth field assessment of foliage blight (Fb4), on a 1–9 scale of increasing resistance, on 11 August 1998, which proved the optimal date for discriminating between clones. The heritability was estimated as 0.878.
2. Glasshouse assessment of tuber blight (Tb %) as a percentage of infected tubers in 1999. The heritability was estimated as 0.870.
3. Whole-plant glasshouse assessment of the presence/absence of Stirling’s major R-gene (R-gene). It was possible to categorise all 190 clones. Further details of this assessment using a simple and complex race are given in Stewart et al. (2003).

Resistance to the white potato cyst nematode *Globodera pallida* (PCN), from Bryan et al. (2004), scored as the count of cysts and analysed after a square root transformation. The heritability was estimated as 0.937.

### Method for QTL mapping

Hackett et al. (2013) have described the theory for constructing a SNP linkage map using dosage information, and for using dosage information in QTL interval mapping, assuming a model of random chromosomal segregation. The QTL mapping steps can be summarised as:

1. Estimate the QTL genotype probabilities at each SNP from the parental genotypes and phases and the offspring dosages. This uses a Hidden Markov Model (HMM) and gives a 36 × *s* matrix *P*_G_ for each offspring *G, G* = 1*…n* with the probability of each of the 36 possible QTL genotypes π at the *s* SNPs on a linkage group.
2. Interpolate QTL genotype probabilities between the SNPs at a 1 centiMorgan (cM) spacing along each chromosome, using a cubic smoothing spline.
3. Model the trait values as a function of the QTL genotype, using a normal mixture model with a constant variance.

#### Fitting a normal mixture model to the trait values

Jansen (1992) developed a general mixture model for QTL mapping and showed how the model fitting can be separated into two steps, a weighted regression step where the trait values are regressed on the QTL genotypes, weighted by their probabilities *P*_G_ (derived initially from the HMM in step 1), and an updating step where the QTL genotype probabilities are updated from marker data and the current estimates of the QTL model parameters.

Formally, the likelihood of the trait data $Y_{ 1} \ldots Y_{n}$ and the observed marker data $x_{ 1} \ldots x_{n}$ can be written as

$$L = \prod\limits_{G = 1}^{n} {f(Y_{G},x_{G}) =} \prod\limits_{G = 1}^{n} {p(x_{G})f(Y_{G} |x_{G})}$$

where $f(Y_{G} |x_{G})$ is the probability density function of the trait conditional on the marker data, with parameter vector *θ*.

The maximum likelihood equation is

$$\frac{{\partial \log L_{{}}}}{\partial \theta} = 0$$

and Jansen (1992) showed how the left hand side of this can be rewritten in terms of the inferred QTL genotypes π as

$$\frac{{\partial \log L_{{}}}}{\partial \theta} = \sum\limits_{G = 1}^{n} {\sum\limits_{\pi}^{{}} {p(\pi_{G} |Y_{G},x_{G})} \frac{\partial}{\partial \theta}\left[{\log p(\pi_{G})} \right]} + \sum\limits_{G = 1}^{n} {\sum\limits_{\pi}^{{}} {p(\pi_{G} |Y_{G},x_{G})} \frac{\partial}{\partial \theta}\left[{\log f(Y_{G} |\pi_{G})} \right]}$$

The conditional probabilities $p(\pi_{G} |Y_{G},x_{G}) = p(\pi_{G} |x_{G})f(Y_{G} |\pi_{G})/f(Y_{G} |x_{G}) = P_{G} f(Y_{G} |\pi_{G})/f(Y_{G} |x_{G})$ where *P*_*G*_ are the QTL genotype probabilities from the HMM. The likelihood equation can be solved (Jansen 1992) using an iterative approach based on the EM algorithm, alternating an Expectation step updating the conditional probability with a Maximisation step of a weighted regression to calculate the parameters of the QTL model $f(Y_{G} |\pi_{G})$ until the likelihood converges. The conditional probabilities are the QTL genotype probabilities *P*_G_ from the HMM for the first step, and are then updated using the probability distribution function $f(Y_{G} |\pi_{G})$ and rescaled to sum to one. Our experience with diploid QTL mapping software such as MapQTL 5 (van Ooijen 2004) is that occasionally the number of iterations can be high and spurious QTLs can be inferred in regions where the marker information is sparse: to avoid this here, a maximum of ten iterative steps was imposed. The LOD score is calculated as log_10_(likelihood ratio) = log_10_(*L*) *−* log_10_(*L*_0_), where *L*_0_ is the likelihood of the trait data in the absence of a segregating QTL i.e. that the trait has a normal distribution with a single mean for the population.

An alternative model fitting approach omits the iterative updating of the weights and uses a single regression step, weighted by the QTL genotype probabilities *P*_G_ from the HMM. This approach is used in some diploid QTL mapping software such as the procedures in GenStat 15 for Windows (Payne et al. 2012) and some of the options in *R*/QTL (Broman et al. 2003). For a dense marker map, this should be a good approximation to the iterative procedure. The simulation study compares these two model fitting procedures.

#### The form of the QTL model $f(Y|\pi)$

Kempthorne (1957) discussed the partitioning of the genetic variance of tetraploid individuals in a random mating population at equilibrium and expressed the trait value *Y*_*G*_ of an individual with genotype *A*_*i*_ *A*_*j*_ *A*_*k*_ *A*_*l*_ as

$$Y_{G} = \mu + \alpha_{i} + \alpha_{j} + \alpha_{k} + \alpha_{l} + \beta_{ij} + \beta_{ik} + \beta_{il} + \beta_{jk} + \beta_{jl} + \beta_{kl} + \gamma_{ijk} + \gamma_{ijl} + \gamma_{ikl} + \gamma_{jkl} + \delta_{ijkl}$$

where *µ* is the population mean, *α*_*i*_ etc. are the main effects of the alleles and *β*_*ij*_, *γ*_*ijk*_, and *δ*_*ijkl*_ etc. are diallelic, triallelic and tetra-allelic interactions, respectively. For a full-sib population, let the homologous chromosomes be numbered 1–4 for parent P1, and 5–8 for parent P2. An individual will inherit alleles* A*_*i*_ and* A*_*j*_, 1 ≤ *i* ≤ 3; *i* < *j* ≤ 4 from P1 and* A*_*k*_ and* A*_*l*_, 5 ≤ *k* ≤ 7 and *k* < *l* ≤ 8 from P2. Let *X*_*i*_, *i* = 1…8 be 0/1 indicator variables corresponding to allele* A*_*i*_ absent/present for that individual. As discussed by Hackett et al. (2001), Kempthorne’s model can be rewritten for an F_1_ population as

$$Y_{G} = \mu + \mathop \sum \limits_{i = 1}^{8} \alpha_{i} X_{i} + \mathop \sum \limits_{i = 1}^{7} \mathop \sum \limits_{j = i + 1}^{8} \beta_{ij} X_{i} X_{j} + \mathop \sum \limits_{i = 1}^{3} \mathop \sum \limits_{j = i + 1}^{4} \mathop \sum \limits_{k = 5}^{8} \gamma_{ijk} X_{i} X_{j} X_{k} + \mathop \sum \limits_{i = 1}^{4} \mathop \sum \limits_{k = 5}^{7} \mathop \sum \limits_{l = k + 1}^{8} \gamma_{ikl} X_{i} X_{k} X_{l} + \mathop \sum \limits_{i = 1}^{3} \mathop \sum \limits_{j = i + 1}^{4} \mathop \sum \limits_{k = 5}^{7} \mathop \sum \limits_{l = k + 1}^{8} \delta_{ijkl}.$$

There are too many parameters to be estimated here from the 36 genotype means. As each individual inherits precisely two alleles from each parent, Kempthorne’s model needs to be modified to take into account the constraints that *X*_1_ + *X*_2_ + *X*_3_ + *X*_4_ = 2 and *X*_5_ + *X*_6_ + *X*_7_ + *X*_8_ = 2. One such model, which will be referred to as the additive model, contains only the main effects *α*_*i*_ from Kempthorne’s model. Taking into account the constraints on *X*_*i*_, this has the form

1 $$Y_{G} = \mu_{C} + \alpha_{2} X_{2} + \alpha_{3} X_{3} + \alpha_{4} X_{4} + \alpha_{6} X_{6} + \alpha_{7} X_{7} + \alpha_{8} X_{8}.$$

In this model, the constant *µ*_*C*_ is harder to interpret because of the constraints and does not correspond either to the trait mean or to a specific genotype mean.

An alternative model, which will be referred to as the complete model, fits parameters to all of the 36 possible genotype classes. Let *X*_1256_ etc. be 0/1 indicator variables corresponding to an individual having genotype A_1_A_2_A_5_A_6_ or not, with the constraint that these sum to one. Taking into account this constraint, the model has the form:

2 $$Y_{G} = \mu_{C} + \eta_{1257} X_{1257} +\cdots + \eta_{3478} X_{3478}$$

where the constant *µ*_*C*_ corresponds to the mean for the first genotype A_1_A_2_A_5_A_6_ and the genotype effects η_1257_ etc. are differences from this. This model may enable the detection of dominance effects and other interactions that might be missed by the additive model.

#### Exploration of simpler models

The 36 genotype means obtained from the complete model (2) at the most likely QTL location can be explored to identify whether a simple model with two different QTL alleles fits the means. Possible simple models are:

- Simplex QTL Qqqq × qqqq, which segregates in a 1:1 ratio
- Duplex QTL QQqq × qqqq, either segregating in a 1:4:1 ratio qqqq:Qqqq:QQqq where there is additivity or partial dominance, or 5:1 Q-qq:qqqq where there is complete dominance
- Double-simplex QTL Qqqq × Qqqq, either segregating in a 1:2:1 ratio qqqq:Qqqq:QQqq where there is additivity or partial dominance, or 3:1 Q-qq:qqqq where there is complete dominance

The best model among these can be identified using an information criterion such as the minimum Schwarz information criterion (SIC) (Schwarz 1978): $SIC = - 2\log L + p\log m_{o}$ where *L* is the likelihood for the simple model, *p* is the number of parameters in the simple model and *m*_*o*_ is the number of observations (i.e. the 36 genotype means).

This approach can also be used after fitting the additive model (1). The genotype means at the most likely QTL location *S* are calculated from the probability matrices {*P*_G_} from the HMM. Let *P*_SGk_ *, G* = 1…*n*, *k* = 1…36 be the probability that individual *G* has genotype *k* at position *S*. Then, the QTL genotype means are

3 $$\bar{{Y_{k}}} = \mathop \sum \limits_{G = 1}^{n} P_{SGk} Y_{G}.$$

This strategy fits an additive model, but then tests for non-additive effects at the most likely QTL location.

### QTL simulation study

Hackett et al. (2013) carried out a small study simulating offspring data from the estimated linkage maps to look at the proportion of the offspring genotypes that were estimated correctly by a HMM analysis of the parental genotypes and phases and the offspring dosages. From this study, chromosome XII was picked as a group of short length (87 cM), average SNP density (118 SNPs in the linkage group, or 1.35 markers per cM) and among the best reconstructed (the mean proportion of genotypes estimated correctly was 0.877) to form the basis of the simulation study here. Sets of 200 or 400 offspring were simulated from the parental genotypes and phases for the map of chromosome XII, with an additional marker (the QTL) included at position 27 cM. This marker was only used to simulate trait data and was not used for QTL interval mapping. Different configurations of trait data were estimated for QTL mapping, using the formulae in “Appendix” to estimate the size of allele effect that results in a QTL explaining a given proportion of the trait variance. A single set of SNP data was used for each configuration, and multiple traits were generated within each configuration as a constant *m* (set equal to 10.0), plus the genotype effects, plus a normally distributed term for environmental variation.

The following configurations were simulated. Configurations have 200 offspring and 50 traits in each set unless otherwise stated:

Set 1a: Random data (100 traits)
Set 1b: Random data (100 traits and 400 offspring)
Set 2a: Simplex QTL, expected to explain 15 % of the trait variance, on homologue h1 from parent 1
Set 2b: Simplex QTL, expected to explain 10 % of the trait variance, on homologue h2 from parent 1
Set 2c: Simplex QTL, expected to explain 5 % of the trait variance, on homologue h2 from parent 1
Set 2d: Simplex QTL, expected to explain 5 % of the trait variance, on homologue h2 from parent 1, using 400 offspring
Set 3a: Duplex additive QTL, expected to explain 10 % of the trait variance, on homologues h6 and h8 from parent 2
Set 3b: Duplex dominant QTL, expected to explain 10 % of the trait variance, on homologues h1 and h2 from parent 1

The traits in each of these sets were analysed using the additive model (1) to find the location with the highest LOD score on chromosome XII and to see how well the QTL configuration was reconstructed. The random data (set 1) and the three sets with a QTL explaining 10 % of the trait variance were also analysed using the complete model (2). In both cases, the results from fitting the model as a single weighted regression were compared to fitting the model by an iteratively reweighted regression process. The three sets with a QTL explaining 10 % of the trait variance were analysed further to identify whether there were two-allele models compatible with the genotype means.

A permutation test approach (Churchill and Doerge 1994) was used to establish LOD thresholds for declaring a QTL present. Rather than using a fixed number of permutations, the sequential method of Nettleton and Doerge (2000) was used to obtain confidence intervals for the permutation threshold as follows. Let the maximum LOD scores (ML) from *N* permutations be ordered from smallest to largest, $ML_{(1)} \le\ldots \le ML_{(N)}$. The level α threshold is estimated as $ML_{{\left\lceil {N(1 - \alpha)} \right\rceil}}$, where $\left\lceil j \right\rceil$ denotes the smallest integer greater than or equal to *j*. An approximate 100 (1 − γ) % confidence interval for the threshold is given by ($ML_{(L)},ML_{(U)}$) where

$$L = \left\lceil {N(1 - \alpha) - (\Phi^{- 1} (1 - \gamma/2))\sqrt {N\alpha (1 - \alpha)}} \right\rceil$$

$$U = \left\lceil {N(1 - \alpha) + (\Phi^{- 1} (1 - \gamma/2))\sqrt {N\alpha (1 - \alpha)}} \right\rceil$$

where Φ is the distribution function for the standard normal distribution. If the maximum LOD score for a particular trait is greater than the upper bound $ML_{(U)}$, it is declared that a significant QTL is present, while if it is lower than the lower bound $ML_{(L)}$ then it is declared that there is no QTL present. If any test statistics remain unresolved, further permutations can be run to reduce the size of the confidence interval.

Here, we ran a minimum of 200 permutations for each scenario. From the above equations, the approximate 95 % confidence interval is then given by the *L* = 184th and the *U* = 197th ordered maximum LOD scores. A further 300 permutations were analysed if necessary, and the 465th and the 484th ordered values of the combined sets were used as the 95 % confidence interval. For the simulated sets, the permutations were obtained as four permutations of each of the 50 traits in each set, which were analysed to give a single threshold for each set.

## Results

### Simulation study

#### Analysis of simulation set 1: random data

Table 1 summarises the results of analysing 100 traits generated for a population of size 200 using a normal distribution with a mean *m* of 10 and a standard deviation of one, for the additive and the complete models. The estimated positions had a mean close to the centre of the chromosome but showed a high variability, as expected when no true QTL was simulated. The constant *µ*_*C*_ was estimated close to the simulated values of 10 in all cases. The residual mean square (rms) was close to the simulated value of one except for the complete model with iteration, where it was under-estimated. The LOD score and % variance explained (measured as the adjusted *R* ^2^) were slightly higher with iteration than without for the additive model, and substantially higher for the complete model. The LOD scores were much higher under the complete model due to the high number of parameters being fitted here. Table 2 gives the corresponding results for a population of size 400, which showed similar trends but less inflation of the LOD score and % variance with iteration.

**Table 1:** Simulation set 1a

Parameter	Additive model				Complete model			
	With iteration		Without iteration		With iteration		Without iteration	
	Mean	SD	Mean	SD	Mean	SD	Mean	SD
Position	41.0	29.44	39.9	26.84	39.8	28.54	35.9	16.3
*µ*_*C*_	10.0	0.44	10.0	0.39	10.2	0.62	10.2	0.49
*R* ^2^	3.9	2.34	2.1	1.67	15.9	6.67	2.9	3.12
LOD	2.5	0.81	2.4	0.79	11.3	1.92	9.5	1.74
rms	0.96	0.095	0.98	0.095	0.84	0.114	0.98	0.106

Summary statistics for the peak of the LOD profile of 100 *N*(10,1) traits for a population of size 200, using the additive and complete models. The means and standard deviations (SD) for the position (in cM) of the largest LOD score, the model constant *µ*_*C*_, the % variance explained (*R* ^2^), the LOD score and the residual mean square (rms)

**Table 2:** Simulation set 1b

Parameter	Additive model				Complete model			
	With iteration		Without iteration		With iteration		Without iteration	
	Mean	SD	Mean	SD	Mean	SD	Mean	SD
Position	41.2	26.46	40.4	23.41	43.5	28.8	38.4	17.66
*µ*_*C*_	10.0	0.28	10.0	0.26	10.0	0.39	10.0	0.31
*R* ^2^	1.6	1.08	0.94	0.775	8.1	4.21	1.3	1.27
LOD	2.3	0.78	2.3	0.77	10.7	1.63	9.5	1.41
rms	0.98	0.066	0.99	0.066	0.91	0.066	0.98	0.068

Summary statistics for the peak of the LOD profile of 100 *N*(10,1) traits for a population of size 400, using the additive and complete models

#### Analysis of simulation set 2: simplex QTLs, using the additive model

Table 3 summarises the results of simulation set 2a, with a single simplex QTL explaining 15 % of the variation at a position of 27 cM on homologue h1, chromosome XII, of parent 1. Data were simulated with offspring with homologue h1 (genotype Qqqq) expected to have a mean trait value of *m* + *a* and offspring without homologue h1 (genotype qqqq) expected to have a mean trait value of *m*, where *m* = 10 and *a* = 0.838 (“Appendix”). Because of the constraints in Eq. (1), the expected coefficients are an overall constant *µ*_*C*_ equal to *m* + 2*a*, coefficients equal to $- a$ for α_2_, α_3_ and α_4_ and coefficients of zero for α_6_, α_7_ and α_8_. The means in Table 3 showed good agreement with these. In all simulations, the coefficients for α_2_, α_3_ and α_4_ were significantly less than zero. Four of the 50 simulations showed an (incorrect) significant association with one or more of the homologues from parent 2 when the model was fitted without iteration, and five showed such an association when fitted with iteration. The mean *R* ^2^ was close to the simulated figure of 15 %, and the position was close to that simulated. The true QTL position lay in a one-LOD support interval about the LOD maximum for 47 of the traits, and in a two-LOD support interval for all 50 traits. Two hundred permutations were run for this data set. The LOD scores, both with and without iteration, were above the upper bound of the permutation threshold for all but one trait of the 50 simulated, and below the lower bound for the remaining trait. For this simulation, the QTL was consistently detected, with good agreement with the simulated values.

**Table 3:** Simulation set 2a

Parameter	With iteration		Without iteration	
	Mean	SD	Mean	SD
Position	28.5	4.80	28.4	4.75
*µ*_*C*_	11.64	0.291	11.59	0.266
α_2_	−0.87	0.172	−0.83	0.162
α_3_	−0.85	0.172	−0.82	0.160
α_4_	−0.84	0.164	−0.80	0.153
α_6_	0.02	0.182	0.02	0.158
α_7_	0.07	0.195	0.06	0.183
α_8_	0.00	0.180	0.01	0.163
*R* ^2^	15.8	4.64	14.0	4.17
LOD	8.4	2.25	8.3	2.23
rms	0.99	0.094	1.01	0.094
One (two) LOD support interval	47 (50)		47 (50)	
LOD permutation threshold (*N* = 200)	3.85 (3.48, 4.15)		3.54 (3.25, 4.01)	

Summary statistics for the peak of the LOD profile of 50 traits simulated with a simplex QTL explaining 15 % of the trait variance at 27 cM on homologue h1 of parent 1, using the additive model. This shows the means and standard deviations (SD) for the position (in cM) of the largest LOD score, the model constant *µ*_*C*_, the estimated chromosome effects (α_2_, α_3_ and α_4_ for parent 1 and α_6_, α_7_ and α_8_ for parent 2), the % variance explained (*R* ^2^), the LOD score and the residual mean square (rms). One (two) LOD support interval shows the number of traits for which the true QTL location is in the one-LOD support interval, with the corresponding figures for the two-LOD support interval in brackets, and the final row shows the 95 % permutation threshold and its approximate 95 % confidence interval

Table 4 summarises the results of simulation set 2b, with a single simplex QTL explaining 10 % of the variation at a position of 27 cM on homologue h2, chromosome XII of parent 1. Data were simulated with offspring with homologue h2 (genotype qQqq) expected to have a mean trait value of *m* + *a* and offspring without homologue h2 (genotype qqqq) expected to have a mean trait value of *m*, where *m* = 10 and *a* = 0.665 (“Appendix”). For this simulation set, the expected coefficients are an overall constant *µ*_*C*_ equal to *m*, a coefficient of *a* for α_2_ and coefficients of zero for the other homologues. The means in Table 4 showed good agreement with these for the simulation without iteration. However, the mean *R* ^2^ when fitting the model with iteration was slightly higher than simulated, at 12.0 %. The true QTL position lay in a one-LOD support interval about the LOD maximum for 40 of the traits, and in a two-LOD support interval for 47 traits when analysed without iteration and 48 traits with iteration.

**Table 4:** Simulation set 2b

Parameter	With iteration		Without iteration	
	Mean	SD	Mean	SD
Position	30.0	10.85	31.1	9.03
*µ*_*C*_	9.93	0.365	9.95	0.327
α_2_	0.70	0.205	0.67	0.192
α_3_	0.02	0.230	0.02	0.217
α_4_	0.00	0.186	0.00	0.173
α_6_	0.02	0.231	0.02	0.213
α_7_	−0.01	0.223	−0.01	0.208
α_8_	0.02	0.222	0.02	0.200
*R* ^2^	12.0	4.91	10.2	4.06
LOD	6.5	2.16	6.4	2.12
rms	0.98	0.118	1.00	0.119
One (two) LOD support interval	40 (48)		40 (47)	
LOD permutation threshold (*N* = 200)	3.92 (3.61, 4.57)		3.87 (3.47, 4.47)	
LOD permutation threshold (*N* = 500)	3.96 (3.70, 4.30)		3.87 (3.57, 4.13)	
No. significant traits	42		43	
Position	29.4	11.37	30.8	9.22
α_2_	0.74	0.195	0.70	0.182
*R* ^2^	13.3	4.12	11.2	3.30
rms	0.97	0.120	0.99	0.120
One (two) LOD support interval	32 (40)		33 (40)	

Summary statistics for the peak of the LOD profile of 50 traits simulated with a simplex QTL explaining 10 % of the trait variance at 27 cM on homologue h2 of parent 1, using the additive model. The notation is the same as for Table 3. At the bottom of the table are the values for the position, α_2_, *R* ^2^, the rms and the number of traits where the true QTL location is in the one (two) LOD support interval for the traits above the upper bound of the permutation threshold interval based on 500 permutations

Two hundred permutations were run initially for this data set. The LOD scores were above the upper bound of the permutation threshold for 39 traits, with and without iteration, and below the lower threshold for four traits without iteration and three traits with iteration. A further 300 permutations were run to try to resolve the remaining traits. Table 4 shows the thresholds and confidence limits for both the original 200 permutations and the combined set of 500 permutations, which decreases the size of the confidence interval. Based on 500 permutations, 43 were above the upper threshold without iteration, four were below the lower threshold and three were still unresolved. The corresponding figures with iteration were 42 traits above the upper threshold, four below and four unresolved. We focus here on the traits above the upper threshold. α_2_ was the only significant coefficient for 30 of the 43 traits above the threshold without iteration, and for 29 of the 42 traits above this threshold with iteration: the remainder identified additional homologues as making a significant contribution. The means for the significant traits, at the bottom of Table 4, showed higher values of α_2_ and *R* ^2^ and a lower rms, especially using the model with iteration.

Table 5 summarises the results of simulation set 2c, which is similar to set 2b but with *a* = 0.458, giving a simplex QTL explaining 5 % of the variation. As in simulation 2b, the mean coefficients and rms were estimated accurately without iteration but *R* ^2^ was over-estimated when the model is fitted with iteration. The standard deviation of the estimated position was much higher than for the traits explaining more of the variation. The true QTL position lay in a one-LOD support interval about the LOD maximum for 34 of the traits analysed without iteration and 32 of the traits with iteration, and in a two-LOD support interval for 48 traits when analysed with or without iteration. Five hundred permutations were run for this data set. There were 29 traits above the upper threshold without iterations, 15 below the lower threshold and six were still unresolved. The corresponding figures with iterations were 28 traits above the upper threshold, 15 below and seven unresolved. For the traits above the upper threshold, the size and variance explained were over-estimated, again particularly for the model with iteration. This is an example of the widely reported ‘Beavis effect’ (Beavis 1994; Utz and Melchinger 1994; Xu 2003), where the effects of small QTLs are increasingly over-estimated at lower power. The coefficient α_2_ was the only significant coefficient for 13 and 12 of the traits above the upper threshold without iteration and with iteration, respectively: the remainder either identified additional homologues as making a significant contribution or did not identify α_2_ as significant.

**Table 5:** Simulation set 2c

Parameter	With iteration		Without iteration	
	Mean	SD	Mean	SD
Position	31.1	18.73	31.3	18.28
*µ*_*C*_	10.0	0.507	10.0	0.453
α_2_	0.445	0.269	0.424	0.241
*R* ^2^	7.4	2.85	5.8	2.47
LOD	4.3	1.22	4.2	1.22
rms	0.97	0.088	0.99	0.086
One (two) LOD support interval	32 (48)		34 (48)	
LOD permutation threshold (*N* = 500)	4.11 (3.85, 4.29)		3.95 (3.77, 4.11)	
No. significant traits	28		29	
Position	28.5	17.65	28.5	17.34
α_2_	0.520	0.215	0.483	0.203
*R* ^2^	9.3	1.72	7.4	1.53
rms	0.96	0.094	0.98	0.093
One (two) LOD support interval	17 (26)		19 (27)	

Summary statistics for the peak of the LOD profile of 50 traits simulated with a simplex QTL explaining 5 % of the trait variance at 27 cM on homologue h2 of parent 1, using the additive model. The coefficients for α_3_–α_8_ all have means close to zero and for brevity have been omitted. At the bottom of the table are the values for the position, α_2_, *R* ^2^, the rms and the number of traits where the true QTL location is in the one (two) LOD support interval for the traits above the upper bound of the permutation threshold interval based on 500 permutations

Table 6 explores the effect of increasing the population size from 200 to 400 on the detection of a simplex QTL explaining 5 % of the variation. This improved the precision with which the position, the overall constant *µ*_*C*_, the coefficient α_2_ and the rms are estimated. *R* ^2^ was closer to the expected value of 5 %, especially for the simulations with iteration. The true QTL position lay in a one-LOD support interval about the LOD maximum for 43 of the traits analysed without iteration and 42 of the traits with iteration, and in a two-LOD support interval for 50 traits when analysed without iteration and 49 analysed with iteration. Five hundred permutations were run for this data set. The LOD scores were above the upper permutation threshold for 41 traits, below the lower threshold for six and unresolved for three traits, with or without iteration, compared to 28 or 29 above the upper threshold in the smaller population. There is also less tendency to over-estimate the size of the significant QTLs. The coefficient α_2_ was the only significant coefficient for 26 of the 41 traits above the randomisation threshold without iteration and for 22 of the 41 above the threshold with iteration. The remainder generally identified additional homologues as making a significant contribution; only two in each case did not identify α_2_ as significant.

**Table 6:** Simulation set 2d

Parameter	With iteration		Without iteration	
	Mean	SD	Mean	SD
Position	26.3	11.59	27.0	10.62
*µ*_*C*_	9.9	0.277	9.9	0.235
α_2_	0.488	0.149	0.461	0.136
*R* ^2^	6.1	2.41	5.1	2.25
LOD	6.3	2.10	6.2	2.12
rms	0.98	0.063	0.99	0.061
One (two) LOD support interval	42 (49)		43 (50)	
LOD permutation threshold (*N* = 500)	3.87 (3.72, 4.25)		3.74 (3.60, 4.18)	
No. significant traits	41		41	
Position	24.5	7.58	25.4	6.86
α_2_	0.513	0.149	0.488	0.130
*R* ^2^	6.8	1.98	5.8	1.88
rms	0.98	0.063	0.99	0.061
One (two) LOD support interval	33 (40)		35 (41)	

Details are as for set 2c, but with 400 individuals. Summary statistics for the peak of the LOD profile of 50 traits simulated with a simplex QTL explaining 5 % of the trait variance at 27 cM on chromosome 2 of parent 1, using the additive model. The coefficients for α_3_–α_8_ all have means close to zero and for brevity have been omitted. At the bottom of the table are the values for the position, α_2_, *R* ^2^, the rms and the number of traits where the true QTL location is in the one (two) LOD support interval for the traits above the upper bound of the permutation threshold interval based on 500 permutations

#### Analysis of simulation set 3: duplex QTLs, using the additive model

Table 7 summarises the results of simulation set 3a, with an additive duplex QTL simulated at 27 cM on homologues h6 and h8 of parent 2, chromosome XII, i.e. offspring have expected means of *m*, *m* + *a* or *m* + 2*a* according to whether they inherit neither, one or both of homologues h6 and h8. The QTL effect was set as *a* = 0.576, corresponding to an expected variation explained of 10 %, and *m* = 10. For this simulation set, the expected coefficients are an overall constant *µ*_*C*_ equal to *m*, a coefficient of *a* for α_6_ and α_8_ and coefficients of zero for the other homologues. Again, the figures in Table 7 were close to the expected values, with slight over-estimates for the set with iteration. The true QTL position lay in a one-LOD support interval about the LOD maximum for 46 of the traits analysed without iteration and 44 of the traits with iteration, and in a two-LOD support interval for 48 traits when analysed without or with iteration. Five hundred permutations were run for this data set. There were 42 traits above the upper threshold without iterations, five below the lower threshold and three were still unresolved. The corresponding figures with iterations were 40 traits above the upper threshold, five below and five unresolved. α_6_ and α_8_ were the only significant coefficients for 33 of the traits above the upper threshold, both without iteration and with iteration and just one of these coefficients was significant for a further 4 and 1 traits, respectively: the remainder identified additional homologues as making a significant contribution.

**Table 7:** Simulation set 3a

Parameter	With iteration		Without iteration	
	Mean	SD	Mean	SD
Position	26.9	13.10	27.0	12.25
*µ*_*C*_	10.0	0.316	10.0	0.289
α_2_	0.022	0.198	0.016	0.168
α_3_	−0.015	0.204	−0.011	0.189
α_4_	−0.008	0.196	−0.009	0.180
α_6_	0.607	0.195	0.564	0.174
α_7_	−0.009	0.217	0.001	0.192
α_8_	0.628	0.170	0.576	0.158
*R* ^2^	12.4	5.23	9.7	3.86
LOD	6.4	2.07	6.2	2.00
rms	1.00	0.135	1.030	0.131
One (two) LOD support interval	44 (48)		46 (48)	
LOD permutation threshold (*N* = 500)	4.08 (3.96, 4.28)		3.99 (3.84, 4.12)	
No. significant traits	40		42	
Position	27.2	13.59	27.2	12.26
α_6_	0.649	0.188	0.591	0.170
α_8_	0.658	0.167	0.602	0.154
*R* ^2^	13.9	4.58	10.7	3.339
rms	0.98	0.135	1.02	0.129
One (two) LOD support interval	34 (38)		38 (40)	

Summary statistics for the peak of the LOD profile of 50 traits simulated with an additive duplex QTL explaining 10 % of the trait variance at 27 cM on homologues h6 and h8 of parent 2, using the additive model. At the bottom of the table are the values for the position, α_6_, α_8_, *R* ^2^, the rms and the number of traits where the true QTL location is in the one (two) LOD support interval for the traits above the upper bound of the permutation threshold interval based on 500 permutations

Table 8 compares the above simulation with that for a dominant duplex QTL simulated at 27 cM on homologues h1 and h2 of parent 1, chromosome XII, where the offspring have expected means of *m* + *a* if they carry either homologue h1 or homologue h2 from parent 1, and *m* if they carry homologues h3 and h4 from this parent. Setting *a* = 0.894 corresponds to an expected variation explained of 10 % (“Appendix”). The estimated means from fitting the additive model corresponded less well to the simulated values than for the additive duplex SNP, with lower values for the variance explained. The mean position was still close to that simulated, but fewer of the one-LOD support intervals contained the true QTL position, although figures for the two-LOD intervals were similar. The mean coefficients for α_3_ and α_4_ were significantly negative. Five hundred permutations were run for this data set. There were 27 traits above the upper threshold without iterations, 11 below the lower threshold and 12 were still unresolved. The corresponding figures with iterations were 26 traits above the upper threshold, 12 below and 12 unresolved. A further 500 permutations were run, but this did not improve the resolution. Of the significant traits, 21 had significant, negative coefficients for α_3_ and α_4_ only and a further one had significant coefficients for just one of these under the model without iteration. With iteration, 20 traits had significant negative coefficients for α_3_ and α_4_ only and a further one had significant coefficients for only one of these. We conclude that this additive model does less well in the case of a fundamentally non-additive relationship, but that when a significant QTL is detected, the location and homologues of importance are close to the true values.

**Table 8:** Simulation set 3b

Parameter	With iteration		Without iteration	
	Mean	SD	Mean	SD
Position	29.8	16.55	29.4	15.24
*µ*_*C*_	11.5	0.318	11.4	0.302
α_2_	−0.031	0.193	−0.039	0.183
α_3_	−0.592	0.175	−0.555	0.162
α_4_	−0.574	0.172	−0.542	0.160
α_6_	−0.041	0.180	−0.024	0.172
α_7_	−0.062	0.156	−0.041	0.155
α_8_	−0.053	0.207	−0.042	0.197
*R* ^2^	8.5	3.36	7.1	3.24
LOD	4.8	1.58	4.7	1.59
rms	1.03	0.114	1.05	0.115
One (two) LOD support interval	38 (47)		37 (46)	
LOD permutation threshold (*N* = 500)	4.10 (3.85, 4.52)		3.96 (3.76, 4.43)	
No. significant traits	26		27	
Position	25.0	12.66	26.1	11.48
*R* ^2^	10.93	2.572	9.27	2.542
rms	0.999	0.097	1.017	0.099
One (two) LOD support interval	18 (22)		20 (24)	

Summary statistics for the peak of the LOD profile of 50 traits simulated with a dominant duplex QTL explaining 10 % of the trait variance at 27 cM on homologues h1 and h2 of parent 1, using the additive model. At the bottom of the table are the values for the position, *R* ^2^, the rms and the number of traits where the true QTL location is in the one (two) LOD support interval for the traits above the upper bound of the permutation threshold interval based on 500 permutations

#### Analysis using the complete model

The three sets of simulated traits with a QTL explaining 10 % of the trait variation were re-analysed using the complete model, where parameters for the 36 genotype means are fitted. Table 9 compares the main parameter estimates. Fitting the models without iteration gave mean values for *R* ^2^ and the rms close to the simulated values, but when the models were fitted with iteration *R* ^2^ was badly over-estimated and the rms was under-estimated. The true position lay in the one- and two-LOD support intervals for a much lower number of the traits than for the additive model. Five hundred permutations were run for each of these data sets. For the models fitted without iteration, the numbers of traits with the maximum LOD score above the upper permutation threshold were 27, 19 and 24 for sets 2b, 3a and 3b, respectively. These are lower than the corresponding numbers of significant QTLs detected for the additive models of 43, 42 and 27. When so many parameters are being fitted to a relatively small data set, the possibility for over-fitting is high and the higher LOD threshold means that the power to detect QTLs is reduced.

**Table 9:** Analysis of the traits where a QTL explains 10 %, using the complete model

Parameter	With iteration		Without iteration	
	Mean	SD	Mean	SD
Set 2b: simplex QTL				
Position	32.9	20.15	33.5	12.80
*R* ^2^	19.8	6.09	10.1	4.53
LOD	14.5	2.36	13.2	2.27
rms	0.891	0.120	1.00	0.129
One (two) LOD support interval	14 (25)		24 (32)	
LOD permutation threshold (*N* = 500)	14.72 (14.44, 15.50)		12.89 (12.36, 13.23)	
Set 3a: additive duplex QTL				
Position	33.5	20.99	32.8	11.85
*R* ^2^	19.3	6.36	9.7	4.92
LOD	14.3	2.61	13.0	2.50
rms	0.920	0.132	1.03	0.130
One (two) LOD support interval	15 (28)		23 (33)	
LOD permutation threshold (*N* = 500)	14.95 (14.49, 15.43)		13.01 (12.64, 13.44)	
Set 3b: dominant duplex QTL				
Position	31.2	19.87	30.7	9.63
*R* ^2^	18.2	5.93	9.2	3.51
LOD	13.8	1.79	12.7	1.78
rms	0.920	0.109	1.02	0.111
One (two) LOD support interval	20 (34)		19 (29)	
LOD permutation threshold (*N* = 500)	14.41 (14.07, 14.60)		12.68 (12.22, 12.90)	

For brevity the 36 genotype means are omitted

#### Testing for simpler models using the 36 genotype means from the complete model and the additive model

The 36 genotype means from the three sets of simulated traits explaining 10 % of the trait variance were explored to see whether they were compatible with a simpler model with two alleles at the QTL. The means were obtained by two approaches: fitting 36 means using the complete model, or fitting an additive model and then calculating the genotype means from the genotype probabilities at the most likely location using Eq. (3). Due to the over-estimation of *R* ^2^ when the model was fitted with iteration, only the means from the fitting without iteration were used in each case. The results are summarised in Table 10. The proportions of the significant traits for which the correct simple model was selected were similar for the complete and the additive model. The proportion where the correct model was selected was lower for the simplex set 2b than for the duplex sets 3a and 3b, but further inspection of the simplex sets showed that typically the correct model was the second best, with a very similar *SIC* to the selected model.

**Table 10:** Testing for simpler models for the simulation sets explaining 10 % of the trait variance

	Complete model	Additive model
Set 2b: simplex QTL		
Number significant	27	43
Correct model selected (qQqq × qqqq)	16	29
Correct parent selected	4	1
Other simple model selected	6	11
Additive model selected	1	2
Set 3a: additive duplex QTL		
Number significant	19	42
Correct model selected (qqqq × qQqQ, with 1:4:1 ratio)	14	31
Correct parent selected	4	9
Other simple model selected	1	0
Additive model selected	0	2
Set 3b: dominant duplex QTL		
Number significant	24	27
Correct model selected (QQqq × qqqq, with 5:1 ratio)	18	21
Correct parent selected	5	6
Other simple model selected	1	0
Additive model selected	0	0

This shows the number of times that a significant QTL is detected among the 50 simulated traits, and classifies these as: correct simple model selected using the SIC; correct parent selected (ie model includes only alleles from the segregating parent); other simple model selected; or no model fits better than the additive effects of each chromosome

#### Comparison of model fitting with and without iteration

Some further summary measures were investigated to establish the differences between model fitting with and without iteration, and are shown in Online Resource 1. The ratio of the mean LODs for each simulation set is only slightly above one, being in the range from 1.00 to 1.04 for the additive models and 1.10–1.19 for the complete models. There is more change in the mean *R* ^2^, with the ratio varying from 1.13 to 1.86 for the additive models and 1.96–6.23 for the complete models. Plotting the ratio of the maximum LOD with iteration to that without iteration against the position of the maximum LOD with iteration shows that the LOD ratio is close to one when the maximum position is close to the middle of the chromosome but that there is a clear trend for the ratio to increase when the maximum position approaches the ends of the chromosome (Online Resource 2). There are only a few instances of this in Online Resource 2 part (b), which is based on simulation 2d (a simplex QTL explaining 5 % of the variance in a population of 400, detected for 41/50 traits), but more in Online Resource 2 part (a), with no true QTL present, and in Online Resource 2 part (c), where the complete model is fitted. Near the ends of the chromosome, there is inevitably less information about the QTL genotype provided by the marker data, and the weights will be more sensitive to the trait data and prone to over-fit it. The iterative model fitting procedure was always terminated after ten iterations, and a check was made to see how often there was a failure to converge. As detailed in Online Resource 1, for the additive model at most 2 % failed to converge, and these were when testing a position close to the ends of the chromosome (0–4 and 75–87 cM). For the complete model fitted to 200 observations, more than half of the iterative procedures failed to converge. Bartlett’s test of homogeneity of variance was used to compare the variance of position estimates under the two fitting procedures. For the additive models, there was no significant difference, but for the complete models the variance was significantly larger when fitted with iteration, as detailed in Online Resource 1, due to QTLs being incorrectly detected near the ends of the chromosomes [Online Resource 2 part (c)]. We conclude that the iterative fitting method over-fits in situations of low information: close to the ends of the chromosomes, when there is no strong QTL present, or when the number of parameters to be estimated is high as in the complete model. It is a particular problem in the latter case, where particularly few of the two-LOD support intervals contained the true QTL location.

#### Conclusions from the simulation study

We conclude from the simulation study that the optimal strategy is to fit the additive model to each trait as a weighted regression on the genotype probabilities without iteration. This aims to minimise over-fitting to the data. Two-LOD support intervals were found to be a reasonable approximation to a 95 % confidence interval for the true position, while one-LOD intervals were too small to be a reliable indication. The mean trait values associated with the 36 QTL genotypes can then be calculated from the genotype probabilities at the most likely location, and a range of simple two-allele models fitted to identify which models minimise the SIC. This strategy is used in the analysis of the experimental data below.

### Experimental study

The experimental study was based on 190 F_1_ offspring from the cross between Stirling and 12601ab1. To establish a genome-wide threshold, the sequential permutation procedure of Nettleton and Doerge (2000) was used as described above. A minimum of 200 permutations were analysed for each of the experimental traits, using the additive model without iteration for all 12 chromosomes, and the maximum LOD over the 12 chromosomes was calculated. If the LOD score for a trait was within the approximate 95 % confidence interval for the threshold, a further 300 permutations were analysed.

Table 11 gives details of the QTLs detected for each trait, and the corresponding 95 % confidence interval for the LOD threshold. The LOD profiles were inspected to see if multiple QTLs were present on each chromosome, but none were found.

**Table 11:** Estimated QTL locations from analysis without iteration for the phenotypic traits scored on the experimental population

Trait	Chr.	Position (cM) and two-LOD support interval	LOD	LOD threshold 95 % CI	No. permutations	*R* ^2^	Previously detected in this cross?	Simple model and candidate SNPs (if identified)
Flower colour	X	44 (43–46)	37.9	(4.79, 5.09)	500	51.9	Yes (B2008)	Simplex, h6
Flower colour	XI	84 (53–93)	4.9	(4.79, 5.09)	500	7.9	No	Dominant duplex, h6 and h8. Candidate c1_4947
Flower_colour (residual)	XI	69 (51–91)	5.4	(4.79, 5.15)	500	7.9	No	Dominant duplex, h6 and h8. Candidate c1_4947
Mat	V	15 (14–22)	42.3	(4.89, 6.25)	200	57.7	Yes (B2004, B2008)	Simplex on h1, candidate c2_47609 but possible further effect of h5
Ht	V	21 (14–26)	20.0	(4.67, 5.35)	200	35.6	Yes (B2004)	As for Mat
R-gene	XI	84 (81–87)	40.8	(4.72, 5.43)	200	57.7	Yes (B2004)	Simplex, h4 Candidate c2_37342
Fb4	IV	26 (22–30)	19.7	(4.82, 6.01)	200	33.4	Yes (B2004)	Additive duplex for resistance on h1 and h4 Candidate c2_7756
Fb4	V	21 (13–27)	9.6	(4.82, 6.01)	200	17.8	Yes (B2004)	Simplex, h1 Candidate c2_47609
Tb%	V	22 (13–26)	10.6	(4.91, 5.53)	200	20.7	Yes (B2004)	Simplex, h1 Candidate c2_47609
Tb%	IV	25 (20–34)	8.3	(4.91, 5.53)	200	16.3	Yes (B2004)	Additive duplex for resistance on h1 and h4 Candidate c2_7756
PCN	IV	28 (22–34)	16.6	(4.84, 5.83)	200	29.8	Yes (Br2004)	Additive duplex for resistance on h6 and h8
PCN (residual)	XI	22 (14–35)	5.4	(4.86, 5.21)	500	8.6	Yes (Br2004)	Additive duplex for resistance on h7 and h8. Candidate c2_33657

The two-LOD support interval for the position is shown in brackets. *Chr.* Chromosome. LOD threshold 95 % CI give the approximate 95 % confidence interval for the LOD threshold, and No. permutations gives the number of permutations from which the confidence interval is derived. Where a QTL was previously detected in this cross the reference is indicated as B2004, Bradshaw et al. (2004); Br2004, Bryan et al. (2004), B2008, Bradshaw et al. (2008). Simpler models are indicated as homologous chromosomes h1–h4 from Stirling and h5–h8 from 12601ab1. *Mat.* Maturity, *Ht* canopy height, *R-gene* presence/absence of Stirling’s major R-gene, *Fb4* fourth field assessment of foliage blight, *Tb%* glasshouse assessment of tuber blight, *PCN* counts of potato cyst nematode *G. pallida*. (Residual) after a trait name indicates that the residuals have been analysed after regression on the most significant QTL to remove its effect

#### Flower colour

Among the 190 offspring, 100 were scored as 0 = white (like Stirling), 78 as 1 = blue (like 12601ab1) and the remaining 12 were missing (no flowers). When the 0/1 scores were mapped as a quantitative trait, the most significant association was on chromosome X, with the peak LOD of 37.9 at 44 cM, well above the upper permutation threshold for this trait of 5.09. The only significant homologue effect was that of homologue h6, from 12601ab1, suggesting that this QTL is a simplex effect on h6. When two-allele models were fitted to the genotype means, the model of a simplex allele on h6 had the lowest *SIC* of −17.6, although this was only slightly lower than that of the full (additive) model where SIC = −16.4. A further association was also investigated on chromosome XI, with the peak LOD of 4.9. This lies within the 95 % confidence interval for the LOD threshold, based on 500 permutations (4.69, 5.09). Analysis of the residual flower colour after regression on the effect of LG X increased the LOD to 5.4, above the upper permutation threshold for the flower colour residual of 5.15. In both models, homologues h6 and h8 (from 12601ab1) showed significant, similar-sized effects. A simple model with dominant effects of h6 or h8 had the minimum SIC of −33.7 for the residual flower colour, with an additive effect of h6 and h8 being a slightly worse fit with SIC = −30.3.

Flower colour is known to be controlled by interacting genes, and this was explored by some further modelling. A candidate SNP for the effect on chromosome XI is c1_4947, which mapped to 83.6 cM and is duplex with B alleles on h6 and h8. For the effect on chromosome X, there is no simplex SNP on h6 close to 44 cM and so a ‘pseudo-SNP’ was constructed from a pair of SNPs, c2_27827 at 43.7 cM with phase BAAA × BAAA and c2_27806 at 44.3 cM with phase BAAA × BBAA. The recombination frequency calculated directly between these two loci is 0.01, with LOD score 39.2. There should therefore be less than 1 % error in defining a pseudo-SNP as having h6 if the dosage of B alleles at c2_27806 is one greater than the dosage at c2_27827, and lacking h6 otherwise. The pseudo-SNP segregated in a ratio of 94 h6 present to 96 h6 absent. A binary generalised linear model (GLM) with a logit link function was used to model the 0/1 flower colour scores as a function of the pseudo-SNP on chromosome X, the candidate duplex SNP c1_4947 on chromosome XI and their interaction. The interaction was not significant on the scale of the GLM linear predictor, and the duplex SNP showed a dominant presence/absence pattern rather than an additive effect. Table 12 gives the observed and expected proportions of blue flowers for each genotype as predicted by the generalised linear model.

**Table 12:** Two-gene model for the probability of blue flowers

	Chromosome XI, c1_4947 = AAAA			Chromosome XI, c1_4947 = AAB-		
	Count	Observed proportion	Predicted proportion	Count	Observed proportion	Predicted proportion
Chromosome X, h6 pseudo-SNP = AAAA	16	0.000	0.002	80	0.108	0.108
Chromosome X, h6 pseudo-SNP = AAAB	14	0.143	0.141	80	0.907	0.907

The observed proportions of blue flowers in the four genotype categories are compared with the predicted probabilities from a two-gene model of a simplex gene on chromosome X and a dominant duplex gene on chromosome XI. These are hypothesised to be close to the flower colour loci *F* and *P* respectively (see text)

The candidate SNPs on chromosome X, c2_27827 and c2_27806, both map to the same potato genome superscaffold (PGSC0003DMB000000106), at positions chr10:50697563 and chr10:50615253, respectively. This is in fairly close proximity to superscaffold PGSC0003DMB000000008 which contains DArT markers associated with violet flower colour reported by Śliwka et al. (2012). The candidate SNP on chromosome XI (c1_4947, chr11:41448860) maps to genome superscaffold PGSC0003DMB000000017. The inheritance of flower colour is reviewed by van Eck et al. (1993), who mapped the locus *P* involved in blue anthocyanin production to chromosome XI, and the locus *F* for flower-specific expression of the colour to chromosome X in diploid potato. Bradshaw (2006) reviewed the inheritance in tetraploid potato. We postulate that the SNPs detected here are close to the *F* locus on chromosome X and the *P* locus on chromosome XI (although there are some recombinants), that Stirling has the recessive genotype *pppp ffff* and consequently white flowers, that 12601ab1 has genotype *ppPP fffF* and blue flowers and that only the offspring with genotype *ppP*- *fffF* have blue flowers, as in Table 2.2 of Bradshaw (2006). The probability of an offspring inheriting the dominant *P* allele and the *F* allele and therefore having blue flowers is 5/6 × 1/2 = 5/12, giving expected frequencies here of 74 offspring with blue flowers and 104 with white flowers, which agrees well with the observed figures of 78 with blue flowers and 100 with white.

#### Maturity

Bradshaw et al. (2004, 2008) reported a large QTL affecting maturity on chromosome V, with a simplex allele from Stirling explaining 54 % of the phenotypic variance. This QTL was also detected in the current study, with the peak LOD score of 42.3 at 15 cM for the additive model, which explained 57.7 % of the variance. The chromosomal effects suggested that the most significant effect is that of simplex allele Q_S_qqq on homologue h1 of Stirling. Analysis of the QTL genotype means using simple two-allele models showed that a simplex allele on h1 had the lowest SIC of 84.4, compared to SIC = 85.5 for the full (additive) model. The closest candidate SNP with this simplex configuration is c2_47609 at 18 cM, and regression on this SNP genotype explained 55.0 % of the phenotypic variance. The presence of the Q_S_ allele was associated with earlier maturity (mean difference 2.3, SE 0.15). However, the analysis showed further significant effects associated with 12601ab1, suggesting a simplex allele Q_X_ associated with earliness on homologue h5 of 12601ab1 with a smaller effect. Including this in the analysis of the QTL genotype means gave a lower SIC of 73.9. The nearest SNP with this configuration is c1_15292 at 27 cM. This was also significant (*p* < 0.001) when included in a regression model of the maturity scores and increased the percentage variance explained to 61.0 %, with a mean maturity difference of 0.77 (SE 0.14). Marker c2_47609 (chr05:5972404) maps to genome superscaffold PGSC0003DMB000000243 and is ~1.5 Mb from the recently published CDF1 gene (PGSC0003DMG400018408, chr5:4538880-4541736) coding for plant maturity (Kloosterman et al. 2013).

#### Canopy height

Bradshaw et al. (2004) reported that canopy height showed a similar genetic configuration to maturity and this was confirmed here. The peak of the LOD profile was at 21 cM with a LOD of 20.0, and explained 35.6 % of the phenotypic variance. The simplex SNP c2_47609 from h1 of Stirling explained 30.1 % of the variance in height, and the Q_S_ allele was associated with shorter height (mean difference 8.7 cm, SE 0.96). Inclusion of the simplex SNP c1_15292 from h5 of 12601ab1 increased the percentage variance explained to 35.2 %. This allele was also associated with shorter height, with a mean difference of 3.7 cm (SE 0.93). Analysis of the QTL genotype means also confirmed that a model with separate allele effects for h1 and h5 had the minimum SIC. These results suggest that early maturing potatoes have a lower canopy height and begin to senesce, while later maturing ones continue to grow.

#### Blight resistance

Among the 190 offspring, 113 were classified by Stewart et al. (2003) as having Stirling’s major R-gene, and 77 as lacking it. When the presence/absence scores were mapped as a quantitative trait, the most significant association was on chromosome XI, with the peak LOD of 40.8 at 84 cM. The only significant chromosomal effect was that of homologue h4, from Stirling. Analysis of the QTL genotype means showed that a simplex QTL on h4 has SIC = −32.8, considerably lower than the next best model, the full (additive) model, with the SIC = −16.5. There is a candidate simplex SNP qqqQ at this position on h4, c2_37342, which also has the highest association with the R-gene scores using a chi square test of independence (*χ* ^2^ = 106.4 with one degree of freedom). This Q allele is absent in 73 of the 77 offspring without the R-gene and present in 92 of the 113 with the R-gene. SNP marker c2_37342 maps to genome superscaffold PGSC0003DMB000000575 which is on chromosome VI. However, further analysis of the genome sequence flanking this marker shows a very strong BLAST hit to chromosome XI superscaffold PGSC0003DMB000000623 (*P* value 5.7e-11) at position chr11:44363050, only slightly less significant than the value for the ‘correct’ location (4.9e–15). This indicates that for an as yet unexplained reason in the Stirling × 12601ab1 cross, this marker is segregating as a chromosome XI marker. Superscaffold PGSC0003DMB000000623 (chr11: 44,275,526-44,528,585) is adjacent to superscaffold PGSC0003DMB000000017 (chr11: 40,928,095–44,225,525), which harbours a large cluster of NB-LRR genes at the distal end of chromosome XI including closely related homologues of the late blight resistance gene R3a (Huang et al. 2004; Jupe et al. 2012).

Both foliage blight (Fb4) and tuber blight (Tb %) mapped to the same region of chromosome V as maturity and height, as shown by Bradshaw et al. (2004). For Fb4, the peak of the LOD profile was at 21 cM, with a LOD of 9.6, and the additive model explained 17.8 % of the phenotypic variance and for Tb % the peak of the LOD profile was at 22 cM with a LOD of 10.6 and explained 20.7 % of the trait variance. The best genetic model was a simplex allele Qqqq on homologue h1 of Stirling, with the Q allele associated with susceptibility to blight. There was no evidence that alleles from 12601ab1 had a significant effect on these traits. Regression on the candidate simplex SNP c2_47609 at 18 cM explained 18.0 % of the variance of Fb4 with a mean effect of 1.7 (SE 0.27), and 21.6 % of the variance of Tb %, with a mean effect of 27.2 % (SE 3.9).

A further QTL for Fb4 and Tb % was mapped to chromosome IV, as found by Bradshaw et al. (2004). For Fb4, the peak of the LOD profile for the additive model was at 26 cM, with a LOD of 19.7, and explained 33.4 % of the phenotypic variance and for Tb % the peak of the LOD profile was at 25 cM with a LOD of 8.3 and explained 16.3 % of the trait variance. Exploration of different genetic models indicated that the best model was for Stirling carrying a duplex allele QqqQ with the Q alleles associated with resistance on homologues h1 and h4 and the Qqqq and qqqQ offspring having intermediate resistance. This model had SIC = 96.7 for Fb4, compared to the second best *SIC* of 110.5 for a dominant duplex model, and SIC = 291.6 for Tb %, compared to the second best SIC of 305.3 for the full (additive) model. A candidate SNP with this configuration is the duplex SNP c2_7756 at 25 cM. Regression of the trait values on the three genotypes of this SNP explained 43.6 % of the variance in Fb4 and 19.2 % of the variance for Tb %. The means for Fb4 are qqqq = 1.7 (SE 0.29), qqqQ = 5.1 (SE 0.13) and qqQQ = 6.3 (SE 0.28) (where 9 is resistant). The corresponding means for Tb % are 84.1 % (SE 4.97), 57.1 % (SE 2.35) and 38.2 % (SE 5.06), where a low Tb % indicates resistance. SNP marker c2_7756 (chr04:4023794) locates to potato genome superscaffold PGSC0003DMB000000330, which along with adjacent superscaffold PGSC0003DMB000000296 harbours NB-LRR genes, including close homologues of the functional late blight resistance gene R2 (Jupe et al. 2012).

**Fig. 1:**
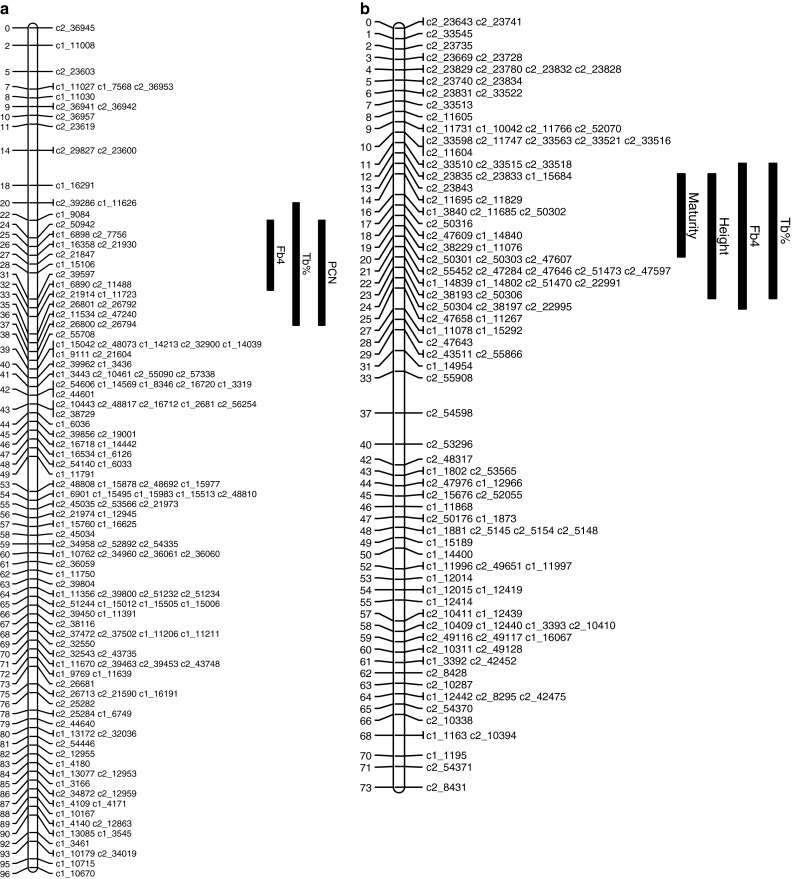

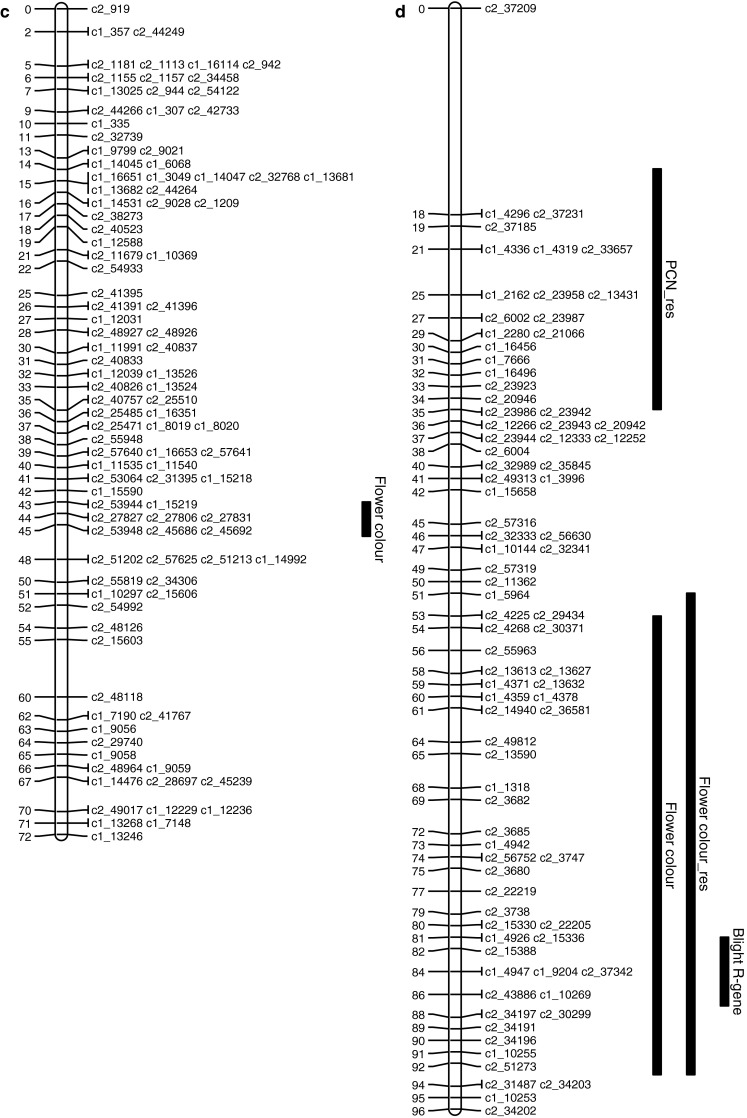
Potato chromosomes showing two-LOD support intervals for QTL locations. **a** Chromosome IV, **b** Chromosome V, **c** Chromosome X, **d** Chromosome XI. The traits are *Fb4* fourth foliage blight score; *Tb* *%* tuber blight, scored as percentage of infected tubers; *PCN* counts of potato cyst nematode *G. pallida*; *PCN_res* residual after regression of PCN on major QTL on LG IV; *flower colour_res* residual after regression of flower colour on major QTL on LG X

Following the analysis of Bradshaw et al. (2004), Fb4 and Tb % were regressed on maturity to remove this effect, and the residuals were analysed. This did not detect further QTLs, or change the positions or inferred models for the QTLs detected on LG IV. This suggests that the QTL on LG IV is a true resistance gene, whereas the QTL on LG V represents the physiological effect of maturity on late blight (Fig. 1).

#### PCN resistance

Bryan et al. (2004) reported a major QTL for PCN resistance on chromosome IV for this population, and this was confirmed here. The peak of the LOD profile for the additive model of the trait √(cyst counts) was at 28 cM, with a LOD of 16.6, and explained 29.8 % of the phenotypic variance. Exploration of different genetic models indicated that the best model is for 12601ab1 carrying a duplex allele qQqQ with the Q alleles associated with resistance (i.e. low cyst counts) on homologues h6 and h8 and the qQqq and qqqQ offspring having intermediate resistance. This had SIC = 74.9, compared with the second best model, full (additive) with SIC = 89.1. There is no candidate SNP with this configuration nearby, and so a pseudo-SNP was constructed from the genotypes of two simplex SNPs c1_16358 (chr04:3240076) at 25.9 and c2_21847 (chr04:5030164) at 26.8 cM. This explained 31.2 % of the phenotypic variance, and the mean √(cyst counts) were qqQQ = 2.9 (SE 0.25), qqqQ = 4.0 (SE 0.11), qqqq = 5.7 (SE 0.21). These SNPs both locate to a region of chromosome IV that contains several homologues of the functional tomato Hero nematode resistance gene and the R2 late blight resistance gene (Ernst et al. 2002; Jupe et al. 2012).

No further QTLs were significant for PCN resistance, but again following Bryan et al. (2004), the residuals after regressing the PCN scores on the pseudo-SNP from chromosome IV were constructed and the QTL analysis was repeated. A further QTL was detected on chromosome XI, as found by Bryan et al. (2004). The peak of the LOD profile for the additive model was at 22 cM, with a LOD of 5.4, and explained 8.6 % of the phenotypic variance. Exploration of different genetic models indicated that the best model was 12601ab1 carrying an additive duplex allele qqQQ with the Q alleles associated with resistance on homologues h7 and h8 and the qqQq and qqqQ offspring having scores midway between the qqqq and the qqQQ individuals (SIC = 81.1, compared with SIC = 87.8 for the second best model, a double-simplex effect). There is a candidate SNP c2_33657 at 21 cM with this duplex configuration on h7 and h8. Regression on this as an additive trait explained 9.7 % of the trait variance, and possession of each Q allele was associated with a mean decrease of 0.7 (SE 0.16) in the PCN score. SNP marker c2_33657 (chr11:2274063) is located in genome superscaffold PGSC0003DMB000000152, which along with adjacent scaffold PGSC0003DMB000000505 contains a small cluster of NB-LRR resistance gene homologues.

## Discussion

In this paper, we have explored and extended the QTL mapping methodology proposed by Hackett et al. (2013) to incorporate information on SNP dosages for QTL mapping. This method is generally applicable to autotetraploid species, provided that the model of random chromosomal segregation is reasonable. Previous work on mapping in autotetraploids, such as Bradshaw et al. (2008), has not been able to align linkage maps from the two parents due to a lack of informative markers segregating in both parents, and consequently QTL mapping has been carried out for each parent separately. Using SNP dosage information, there are far more configurations that enable alignment of the parental maps, and so the effects of alleles from both parents can be studied simultaneously. This gives extra insights, for example that the well-reported maturity effect on chromosome V is not simply derived from the Stirling parent, but that the alleles from the 12601ab1 parent also contribute significantly to this trait. Many QTL mapping methods with sparser maps fit mixture models by iteratively updating the QTL genotype probabilities along the chromosome to allow for the uncertainty between marker positions, but here we have showed that with such a dense map there is no need for such an iterative process, and that using such a process is likely to over-fit the data.

The simulation study also showed that it is better to use an additive model of chromosomal effects to locate the positions most associated with a quantitative trait than to fit a complete model with 36 genotype means at each position. The latter approach again leads to over-fitting to the data. When random traits are analysed by such a flexible model fitted at a large number of positions, high LOD scores are obtained. The LOD threshold therefore has to be set at a high level to avoid false positives, with the consequence that the power to detect QTLs is low. Once the best position has been located using the additive model, the mean trait values for the 36 possible QTL genotypes can be estimated, and explored by fitting a range of two-allele models to see if the data are compatible with a simpler model. We have shown that this approach correctly identifies a dominant duplex QTL in more than half of the significant simulations. The denser maps, with simplex SNPs on almost all the homologous chromosomes and a high density of higher dosage markers, give increased confidence in modelling the allelic effects. It would be possible to recalculate the LOD profile based on a simpler model, but this would require specific programming for each detected QTL, and the simulation study suggests that it would be unlikely to change the QTL location to any large extent.

Despite the advantages of the denser map, the size of the mapping population limits the size of QTLs that can be detected. A population of 180–200 offspring is typical of many experimental studies seen in practice but the simulation study has shown the limitations of this. A single QTL explaining 5 % of the variance in a population of 200 was detected in only 29 of 50 simulations, and the size of the effect was on average over-estimated. If a population of size 400 was used, a QTL explaining 5 % was detected for 41 of 50 simulations, and the magnitude of the effect was estimated more accurately. Larger QTLs can be detected in populations of 180–200, and candidate SNPs for marker-assisted selection can be identified, but to detect smaller QTLs reliably, larger population sizes will be needed.

The simulation study here considered a single QTL together with an environmental effect. In reality, there are likely to be several QTLs of different sizes involved. If a large QTL is detected, its effect can be removed by regression on a candidate SNP and further QTL mapping conducted on the residuals, as was done here for PCN resistance. This should increase the power to detect smaller QTLs. In a clonal crop such as potato, increased replication of the population will increase the heritability and the power to detect smaller QTLs.

The QTL analysis was applied to investigate flower colour, maturity and resistance traits in autotetraploid potato. Most of the QTLs detected here were previously detected using an earlier AFLP and SSR map. The confidence intervals for locations have been improved by the more detailed linkage map, and more information about the genetic model at each QTL becomes available. For several of the QTLs reported here, candidate SNPs can be identified. A major advantage of the SNP map is the ability to link directly to the potato genome sequence for the vast majority of SNP markers used in this study. In several cases, there is strong evidence to suggest that the SNP explaining most of the phenotypic variation is quite close to the causal gene for the trait. For example, for plant maturity, marker c2_47609 maps at most ~1.5 Mb from the gene known to control the trait (Kloosterman et al. 2013). For most of the resistance traits analysed, the best SNP marker maps within NB-LRR gene clusters located at well-documented resistance ‘hotspots’ on chromosomes IV and XI, allowing the possibility of using candidate gene approaches for targeted gene isolation. Moreover, these findings are strongly indicative that the resistances, whether major R gene or partial resistance due to large-effect QTLs, are likely to be due to the action of NB-LRR genes in the potato genome.

### Electronic supplementary material

Below is the link to the electronic supplementary material.

Supplementary material 1 (PDF 19 kb)

Supplementary material 2 (PDF 249 kb)

## Appendix: Relationship between QTL effect and percentage of variance explained

MacKenzie and Hackett (2012) derive general formulae for expected mean squares and heritability as a function of QTL effect and frequency, and these have been adapted here to obtain the relationship between percentage variance explained and QTL effect for different tetraploid configurations. For simplicity, consider a trait controlled by a single bi-allelic QTL, together with environmental variation. Let *f* be the genotype frequency at the QTL, *a* be the genotype effect and *σ* ^*2*^ be the environmental variance in a population of size *n*. Consider a regression on the QTL genotype and partition the total sum of squares (SST) into a sum of squares associated with the genotype (SSG) and a residual sum of squares (SSR).

Applying the formulae of MacKenzie and Hackett (2012) to a QTL with two genotype classes gives the following expected values for the sums of squares: $E\left({SSG} \right) = na^{2} f\left({1 - f} \right) + \sigma^{2}$, with 1 degree of freedom (df). $E\left({SSR} \right) = (n - 2)\sigma^{2}$, with *n* – 2 df. Therefore $E\left({SST} \right) = na^{2} f\left({1 - f} \right) + \sigma^{2} + (n - 2)\sigma^{2} = na^{2} f\left({1 - f} \right) + (n - 1)\sigma^{2},$ with *n* – 1 df.

The expected mean squares* E*(*MSR*),* E*(*MST*) are given by* E*(*SSR*)/(*n* – 2) and* E*(SST)/(*n* − 1).

The expected proportion of variance explained is then calculated as

$$R^{2} = 1 - E(MSR)/E(MST) = \left[{1 - \frac{{\sigma^{2}}}{{\frac{n}{n - 1}a^{2} f\left({1 - f} \right) + \sigma^{2}}}} \right]$$

Or rearranging this

$$a^{2} = \frac{{(n - 1)R^{2} \sigma^{2}}}{{nf(1 - f)(1 - R^{2})}}.$$

For a simplex marker the genotype frequency *f* = 0.5. If the environmental variance *σ* ^*2*^ is set equal to one and the population size *n* = 200, then for *R* ^2^ = 15, 10 and 5 % the corresponding values of *a* are 0.838, 0.665 and 0.458, respectively. For a dominant duplex marker, *f* = 0.83 and *R* ^2^ = 10 % corresponds to a value for *a* of 0.894.

For a duplex marker with an additive effect there are three genotype classes: genotype QQqq with frequency 1/6 and effect +*a*, Qqqq with frequency 4/6 and effect 0 and qqqq with frequency 1/6 and effect –*a.* In this case the expected sum of squares associated with the genotype becomes

$$E\left({SSG} \right) = \frac{{na^{2}}}{3} + \sigma^{2}.$$

The size of the effect *a* can be calculated as above, and is equal to 0.576 for *R* ^2^ = 10 %.

Table 13 compares these calculated values of *a* and *R* ^2^ with those estimated by regressing 100 simulated traits with the calculated values on the true QTL genotype, for each of the simulation scenarios considered in the paper. In each case there is good agreement between the calculated values and those observed from the regression.

**Table 13:** Comparison of the mean and standard deviation (SD) of the estimates of *a* and *R* ^2^ from a regression on true QTL genotype for 100 simulated traits with the intended values

Simulation	Calculated *a*	Estimated *a*	SD	Estimated *R* ^2^	SD
2a, simplex explaining 15 %	0.838	0.827	0.149	14.4	4.54
2b, simplex explaining 10 %	0.665	0.658	0.134	9.46	3.66
2c, simplex explaining 5 %	0.4577	0.448	0.154	4.83	3.20
2d, simplex explaining 5 % in a population of size 400	0.4583	0.463	0.0989	5.05	2.14
3a, additive duplex explaining 10 %	0.576	0.578	0.123	9.94	3.91
3b, dominant duplex explaining 10 %	0.894	0.897	0.172	9.84	3.60
