# Supplementary material for: QTL mapping in autotetraploids using SNP dosage information
Source: Theor Appl Genet. 2014 Jul 1;127(9):1885–904. doi: 10.1007/s00122-014-2347-2 (PMC4145212; doi:10.1007/s00122-014-2347-2)
Supplement: Supplementary file 1 — Supplementary material 1 (PDF 19 kb) [file 122_2014_2347_MOESM1_ESM.pdf]

Table S1 Comparison of simulated sets using models fitted with and without iteration. The column Ratio of the LOD means gives the ratio of the mean LOD score from fitting with iteration to that without iteration. The column Ratio of R^2 means gives the corresponding ratio for R^2. The column Bartlett test statistic for position variance is a test for significant differences in the variance of the position estimates with and without iteration, and its significance compared to a chi-square distribution with one degree of freedom. The % failure to converge shows the percentage of traits (out of the total number of traits × the 88 testing positions along the chromosome) where the iterative approach had not converged after 10 iterations.

Simulation set	True R^2	Model	Pop. size	Ratio of LOD means	Ratio of R^2 means	Bartlett test statistic for position variance	% failure to converge
1a	0%	additive	200	1.04	1.86	0.8^{ns}	0.8
1a	0%	complete	200	1.19	5.48	29.8^{***}	52.2
1b	0%	additive	400	1.00	1.70	1.5^{ns}	0.2
1b	0%	complete	400	1.13	6.23	22.6^{***}	40.7
2a	15%	additive	200	1.01	1.13	0.02^{ns}	2.1
2b	10%	additive	200	1.02	1.18	1.6^{ns}	1.8
2c	5%	additive	200	1.02	1.28	0.03^{ns}	1.8
2d	5%	additive	400	1.02	1.20	0.4^{ns}	0.5
3a	10%	additive	200	1.03	1.28	0.2^{ns}	1.3
3b	10%	additive	200	1.02	1.20	0.3^{ns}	1.4
2b	10%	complete	200	1.10	1.96	9.61^{**}	61.8
3a	10%	complete	200	1.10	1.99	15.1^{***}	56.9
3b	10%	complete	200	1.09	1.98	23.5^{***}	57.8

*** = p < 0.001; **= p < 0.01; ^{ns} = p > 0.05
