# Supplementary material for: QTL mapping in autotetraploids using SNP dosage information
Source: Theor Appl Genet. 2014 Jul 1;127(9):1885–904. doi: 10.1007/s00122-014-2347-2 (PMC4145212; doi:10.1007/s00122-014-2347-2)

Figure S1 Plots of the ratio of maximum LOD score with iteration to that without against the position of the maximum LOD with iteration, for (a) simulation 1a, with no true QTL present, based on the additive model (b) simulation 2d, with a simplex QTL explaining 5% of the trait variance at 27cM in a population of size 400, based on the additive model (c) simulation 3a, with a simplex QTL explaining 10% of the trait variance at 27cM in a population of size 200, based on the complete model.

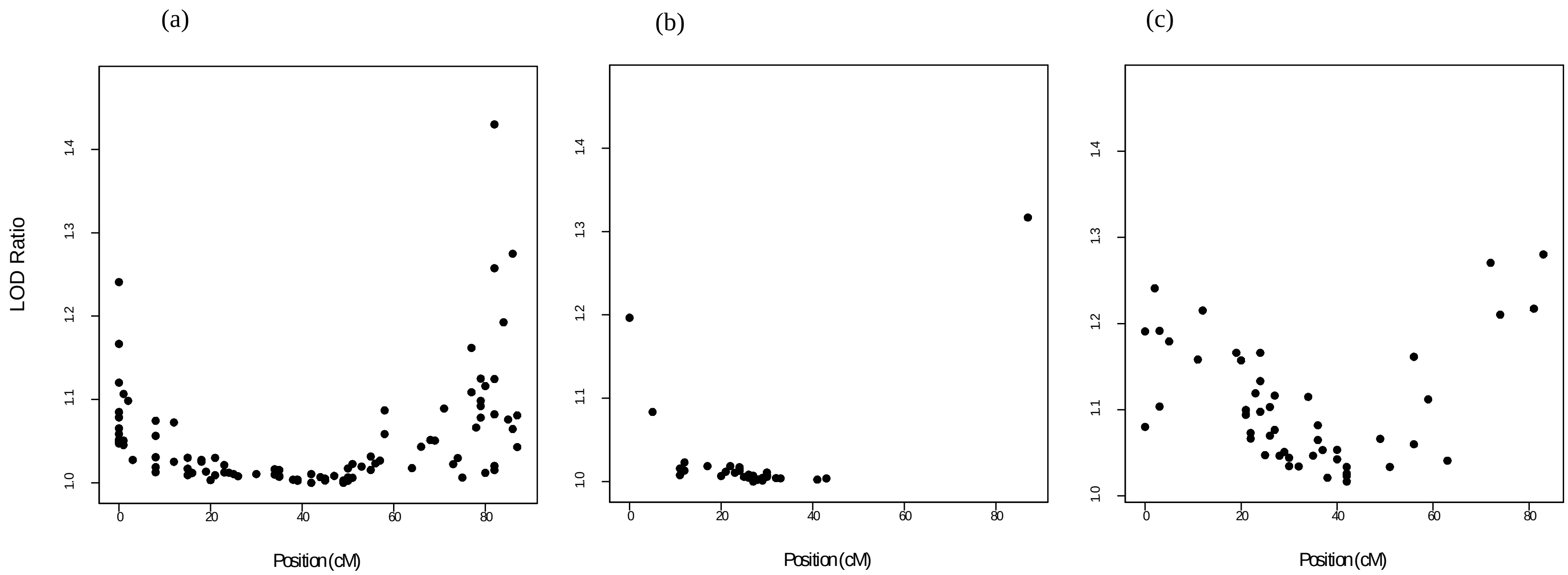

Supplement: Supplementary file 2 — Supplementary material 2 (PDF 249 kb) [file 122_2014_2347_MOESM2_ESM.pdf]
